# Orthogonal plating for complex olecranon fractures: retrospective case series with patient-reported outcomes

**DOI:** 10.1007/s00402-024-05444-w

**Published:** 2024-07-05

**Authors:** Tjalling Aurelius Sebastiaan Ingwersen, Robert Kaspar Wagner, Wouter Veenendaal, Peter Kloen

**Affiliations:** 1 Department of Orthopedic Surgery and Sports Medicine, Amsterdam UMC, location AMC, Meibergdreef 9 AMC / K1 207 Meibergdreef 9, Amsterdam, 1105AZ The Netherlands; 2 Amsterdam Movement Sciences, Musculoskeletal Health, Meibergdreef 9, Amsterdam, The Netherlands

**Keywords:** Fixation, Fracture, Olecranon, Orthogonal, Plate

## Abstract

**Introduction:**

Treatment for complex olecranon fractures with metaphyseal comminution can be challenging. To improve reduction maneuvers and augment stability, we apply a small medial and/or lateral locking compression plate (LCP) prior to placing a posterior contoured 3.5 mm–2.7 mm LCP. The aim is to describe our technique and outcomes of this “orthogonal” plating technique.

**Material and Methods:**

26 patients were treated with orthogonal plating. Clinical outcome variables were available for all patients at a median of 27 months (IQR 6–54), and patient-reported outcomes (Q-DASH and MEPS) for 23 patients at 38 months (IQR 18–71).

**Results:**

All fractures healed at a median of 2.0 months (IQR 1.5–3.8). The median elbow flexion was 120°, extension-deficit 15°, pronation 88°, and supination 85°. The median Q-DASH was 9 (IQR 0–22) and the median MEPS was 90 (IQR 80–100). Hardware was electively removed in seven patients. One patient had a late superficial infection that resolved with hardware removal and antibiotics, and one patient had two consecutive re-fractures after two hardware removals; and healed after the second revision surgery.

**Conclusion:**

Orthogonal plating with a posterior LCP and a small medial and/or lateral LCP is a safe technique that leads to excellent healing rates, and good clinical and patient-reported outcomes.

## Introduction

Olecranon fractures are common injuries and occur in a bimodal distribution. 45% of olecranon fractures are comminuted; and open reduction and internal fixation with plate osteosynthesis has become the standard treatment for these fractures, especially in younger patients [1]. The goal of operative treatment is to restore the articular surface and to obtain a stable elbow allowing early rehabilitation [1, 2].

In our practice, we prefer the use of a single posterior contoured 3.5 mm–2.7 mm locking compression plate (LCP) in comminuted olecranon fractures. We previously reported good results in a retrospective case series; and tested this construct biomechanically [3, 4]. When there is extensive metaphyseal comminution, obtaining a perfect and stable alignment prior to placing the definitive posterior plate can be challenging. To facilitate reduction maneuvers and increase stability, we started adding one or two small 2.0–2.4 mm LCPs on the medial and/or lateral side before placing the posterior LCP; creating an “orthogonal” plate configuration. In our experience, reconstructing the proximal ulna from distal to proximal with these small plates provides a more stable docking site for the most proximal fragment.

Orthogonal plating has been described previously by Morwood et al. in 11 patients with a sagittal olecranon fracture with an intra-articular sagittal split [5]. Contrasting to our strategy, they applied a lateral plate only after placing the posterior plate to augment stability. All fractures healed, and the authors reported good clinical outcomes (flexion-extension: 129° to 24°, pronation-supination: 89° to 79°) and patient-reported outcomes (Disabilities of the Arm, Shoulder, and Hand score: 7). The authors recognized the limitations of the relatively small number of patients and short follow-up of 15 months.

The aim of this retrospective case series was (1) to describe our technique of orthogonal plating for complex olecranon fractures with metaphyseal comminution; and (2) to evaluate clinical (healing, range of motion, and complications) and patient-reported outcomes ([Quick] Disabilities of the Arm, Shoulder and Hand, and the Mayo Elbow Performance Scale).

## Materials and methods

Between July 2012 and March 2022, 110 olecranon fractures were treated by the senior author (a fellowship trained orthopedic traumatologist) and registered in a prospectively kept surgical database. We identified all consecutive patients with an olecranon fracture who were treated with a posterior LCP in combination with a small medial and/or lateral LCP. Twenty-six patients were identified and included in this series. Ethical approval was waived by the local Medical Ethics Review Committee (W23_001 # 23.022). This study was conducted according to the principles of the Declaration of Helsinki and reported using the Strengthening the Reporting of Observational Studies in Epidemiology (STROBE) guideline. Informed consent for study participation was obtained by the treating physician and senior author.

Clinical baseline and surgical variables were collected for all patients from the electronic medical record. Clinical outcomes were available for all patients at a median follow-up of 27 months (IQR 6–54).

All patients were invited to report patient-reported outcome measures (PROMs) that consisted of the Quick Disabilities of the Arm, Shoulder and Hand (Q-DASH), and the Mayo Elbow Performance Scale (MEPS). These outcomes were available for 23/26 patients at a median follow-up of 38 months (IQR 18–71). The remaining 3/26 were lost to follow-up: one due to emigration, one due to psychological problems preventing participation, and one patient did not respond.

The Q-DASH is a validated 11-item score on disabilities and symptoms of the hand, arm and shoulder [6–8]. There are five response options for each item, ranging from no difficulty to inability to perform a given task. The summative score ranges from 0 (no disability) to 100 (the greatest possible disability). The MEPS consists of a 4-item scale on pain (45 points); range of motion (20 points); stability (10 points); and function (25 points). The summative score ranges between 5 and 100, with higher scores indicating better function [9]. All olecranon fractures were categorized using the Mayo classification [10]. The Mayo classification offers a treatment algorithm and prognosis that depends on the specific fracture type. The classification encompasses three types, ranging from Type 1 to Type 3, determined by stability and displacement observed on radiographs. Type 1 fractures are non-displaced, Type 2 fractures are displaced with a stable ulnohumeral joint, and Type 3 fractures are displaced olecranon fractures associated with an unstable ulnohumeral joint and torn collateral ligaments.

### Surgical technique

**Fig. 1:**
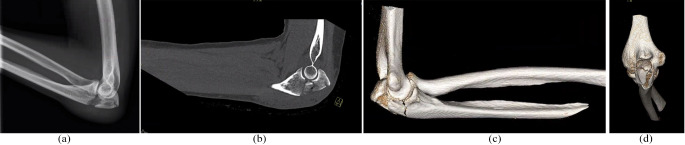
Preoperative imaging of a 47-year-old female with a comminuted and displaced Mayo II-B olecranon fracture (case 24, Table 1). **(a, b)** Lateral radiograph and sagittal computed tomography (CT-) scan imaging; **(c, d)** lateral and dorsal view of a three-dimensional reconstruction of the CT-scan

Orthogonal plating with a small medial and/or lateral 2.0–2.4 mm LCP in addition to the posterior 2.7–3.5 mm LCP was indicated for patients with a complex olecranon fracture with metaphyseal comminution (Fig. 1a-d).

**Fig. 2:**
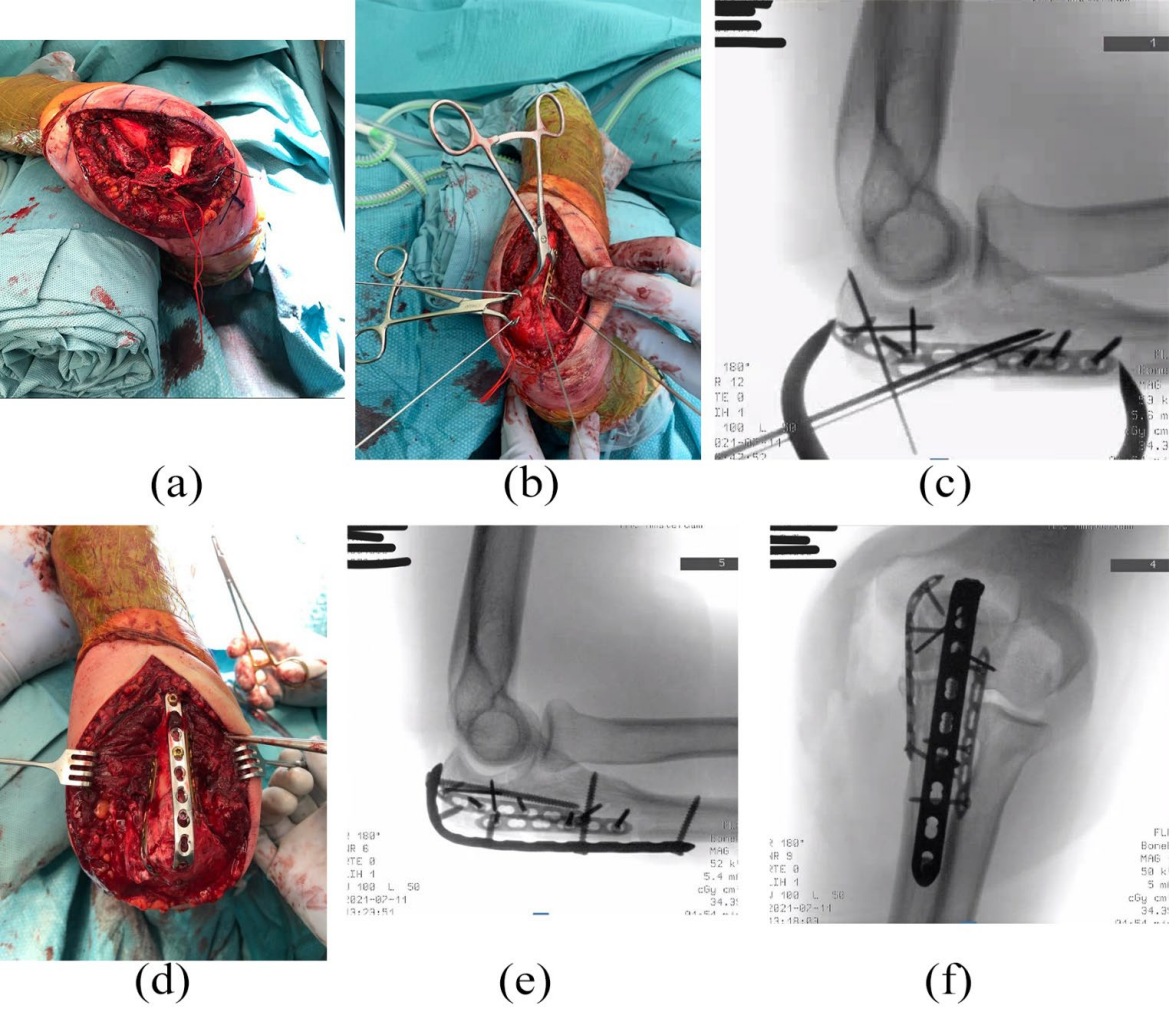
Intraoperative imaging of a 47-year-old female with a comminuted and displaced Mayo II-B olecranon fracture (case 24, Table 1). **(a)** The extended posterior approach is visible. The ulnar nerve is released and protected using a vessel loop, and the proximal olecranon is reflected on the triceps tendon; **(b)** The fracture is reduced and temporarily fixated using clamps and K-wires, and a 2.0 mm locking compression plate (LCP) is applied laterally; **(c)** Fluoroscopic imaging after applying the lateral and medial 2.0 mm LCPs; **(d)** The posterior 2.7 mm LCP is bent proximally, slid under the triceps tendon, and then fixated using unicortical locking screws proximally and bicortical standard screws distally. **(e, f)** Lateral and anteroposterior fluoroscopic imaging displaying the final reduction

A pre-operative CT-scan was made in 13/26 patients. All patients were operated in supine position with the injured arm draped over the chest. A single posterior approach was used in all patients. The posterior approach is a standard posterior longitudinal incision starting at the crista of the proximal ulna reaching to the most proximal palpable point of the olecranon with a small curve over the tip of the olecranon on the radial side to prevent damage to the ulnar nerve. Proximally the incision is extended approximately 5 cm. The ulnar nerve is released and protected in case of a large medial fragment or need for direct visualization of the medial side of the proximal ulna. The proximal olecranon fragment is rotated proximally on the triceps tendon (Fig. 2a). We use the traumatic interval between the lateral aspect of the proximal ulna and the anconeus muscle (Boyd approach) if the radial head is fractured and needs to be replaced or fixated [11]. The fracture is then reduced from deep to superficial and from distal to proximal. The fragments are temporarily transfixed using 1.25–1.6 mm Kirschner wires, which are then replaced by small medial and/or lateral augmentation plates (Fig. 2b and c). Ideally we use standard (non-locking) screws in the small (medial and/or lateral) mini-plates so that we have bicortical purchase. We use 2.0 mm and/or 2.4 mm LCPs (dePuy Synthes, Amersfoort, The Netherlands) of different lengths. No compression is used, as these plates function merely to keep the cortical fragments buttressed in their anatomic place as “pieces in the puzzle”. Impaction of the articular surface is corrected with a thin-bladed chisel using the distal humerus as a template. To support articular disimpaction, we used cancellous allograft chips in 7/26 patients. Cortical defects were filled using an iliac crest bone graft in 2/26 patients, demineralized bone matrix (DBX, DePuy Synthes, Amersfoort, The Netherlands) in 4/26 patients, and cancellous bone from the removed radial head in one patient.

Once the shaft and proximal metaphysis of the ulna are rebuilt, the reduction of the articular surface is completed by reducing the most proximal olecranon fragment with the attached triceps tendon by extending the elbow. Temporary fixation is provided by 1.6 mm K-wires and the reduction is evaluated fluoroscopically.

Then, the posterior plate is contoured and placed. The type of plate used for fixation depends on the morphology of the fracture. The most commonly used posterior plate was the straight 3.5 mm LCP (7/26) (DePuy Synthes, Amersfoort, The Netherlands) that is bent proximally to create a “hook” over the olecranon (Fig. 2d). In smaller patients, we used a 2.7 mm straight LCP. We rarely use the pre-shaped olecranon plate for posterior fixation, although others have reported good results [12]. The most proximal part of the posterior plate is slid under the triceps after a stab incision is made. In the proximal aspect unicortical locking screws are used to not penetrate the articular surface of the trochlea. Distally, we use bicortical standard screws. For the long intramedullary or oblique screw, a 3.5 mm standard screw is used. The hole for this screw is made with an oscillating drill that allows gentle passage of the drill without creating a fausse route. Occasionally the drill encounters a more distal screw. Retracting the drill a few millimeters and then advancing again using the oscillating feature on the drill, will allow clearance of the pathway. Anatomic alignment is confirmed by fluoroscopic evaluation (Fig. 2e and f). Magnification of the view allows careful inspection of the joint in the lateral view. Imperfections should be revised.

In 7/26 patients there was an associated radial head fracture, this was treated in four different ways. One was managed non-operatively, one fracture was treated with K-wires, two were managed with screw fixation, and in three patients a radial head prosthesis was placed (Wright Evolve Radial Head prosthesis, Wright Medical, Amsterdam, The Netherlands). A lasso suture (Tie-Cron 2, Dublin, Ireland) was placed around a coronoid fracture fragment and diverted dorsally via two predrilled tunnels in two patients.

**Fig. 3:**
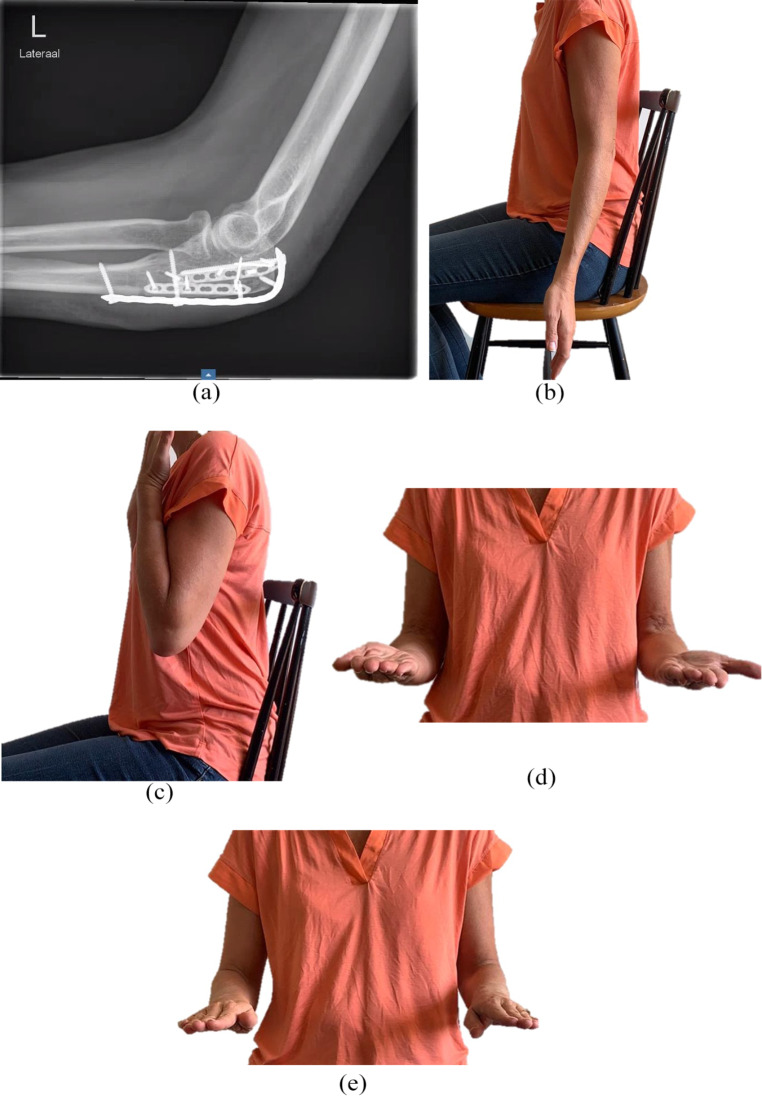
Postoperative imaging of a 47-year-old female with a comminuted and displaced Mayo II-B olecranon fracture (case 24, Table 1). **(a)** Lateral radiograph 6 weeks postoperatively; **(b, c, d, e**) Range of motion at two years postoperatively, showing only a small extension deficit

The elbow is then taken through a gentle range of motion. The ulnar nerve is not transposed. A posterior splint is applied for 7–10 days to allow wound healing. Afterwards, gentle active assisted range of motion exercises are started. Clinical and radiographic follow-up is done postoperatively and after six and 12 weeks or until healing (Fig. 3a-e).

### Statistical analysis

Categorical variables are presented as frequencies. Numerical variables are presented as medians with interquartile range (IQR). Missing data were accounted for by pairwise deletion (complete case analysis). Data from the electronic medical record were collected using a Microsoft Excel spreadsheet. Patient-reported outcomes were collected using a paper data collection sheet. All statistical analyses were performed using R version 4.3.0 [13].

## Results

### Clinical baseline and surgical variables (*n* = 26)

There were 12 males and 14 females with a median age of 55 years (IQR 47–69), see Table 1. There were 24/26 acute fractures, one fracture that was 16 days old, and one fracture that was eight months old (due to an earlier misdiagnosis). The dominant arm was involved in 9/26 patients. The mechanism of injury was a fall from standing height in 9/26 patients, a fall from height in 4/26, and a traffic accident in the remaining 13/26 patients.

**Table 1:** Characteristics and outcomes of individual patients. “-“ = missing or not applicable; LCP = locking Compression plate; Q-DASH = quick disabilities of the arm, shoulder and hand; MEPS = Mayo Elbow Performance scale; ROM = range of motion; PROMs = patient-reported outcome measures

Patient	Age and Sex	Energy mechanism	Mayo	Number of fragments	Posterior plate	Lateral plate	Medial plate	Q-DASH	MEPS	ROM	Time to consolidation (months)	Time to removal (months)
1	71 M	High	2B	≥ 5	LCP 2.7 mm	2.4 mm reconstruction	2.4 mm reconstruction	7	100	55°	1.5	-
2	31 M	Low	3B	≥ 5	LCP 3.5 mm pelvic reconstruction plate	LCP 2.0 mm, LCP 2.0 mm	LCP 2.0 mm	23	85	110°	3.0	-
3	69 M	Low	2B	≥ 5	LCP 3.5 mm pelvic reconstruction plate	LCP 2.7 mm	LCP 2.7 mm	53	75	65°	5.5	30
4	43 F	Low	3B	3	LCP 3.5 mm pelvic reconstruction plate	-	LCP 2.5 mm (Compact Hand Module)	16	95	120°	6.0	10
5	73 M	Low	2B	3	LCP 3.5 mm pelvic reconstruction plate	-	LCP 2.0 mm	0	100	80°	2.0	-
6	79 F	Low	2B	2	LCP 3.5 mm pelvic reconstruction plate	LCP 2.0 mm	-	39	90	105°	2.0	-
7	54 M	High	2B	3	LCP 3.5 mm pelvic reconstruction plate	LCP 2.0 mm	-	55	75	105°	5.0	11
8	58 F	Low	2B	4	LCP 3.5 mm pelvic reconstruction plate	LCP 2.0 mm	LCP 2.0 mm	9	85	125°	5.0	9
9	68 F	Low	3B	4	LCP 3.5 mm pelvic reconstruction plate	LCP 2.4 mm	2.4 mm pelvic	55	40	75°	1.5	-
10	69 M	Low	3B	≥ 5	LCP 3.5 mm pelvic reconstruction plate	LCP 2.0 mm	T-plate 2.0 mm	15	90	95°	4.0	-
11	51 M	Low	2B	2	LCP 3.5 mm pelvic reconstruction plate	LCP 2.7 mm	-	0	100	135°	6.5	-
12	37 M	Low	2B	≥ 5	LCP 3.5 mm pelvic reconstruction plate	-	LCP 2.0 mm	21	65	115°	2.5	-
13	72 F	Low	3B	3	LCP 3.5 mm pelvic reconstruction plate	LCP 2.0 mm	-	5	100	100°	2.0	-
14	48 M	Low	2B	3	LCP 3.5 mm pelvic reconstruction plate	-	LCP 2.5 mm (Compact Hand Module)	0	100	110°	6.0	6
15	27 M	Low	2 A	2	LCP 2.7 mm	-	LCP 1.5 mm (Compact Hand Module)	0	100	115°	1.5	7
16	44 F	High	2B	2	LCP 3.5 mm pelvic reconstruction plate	-	LCP 2.4 mm	2	100	125°	2.0	12.5
17	65 F	High	2B	≥ 5	LCP 3.5 mm pelvic reconstruction plate	LCP 2.0 mm	LCP 2.0 mm	5	100	110°	1.0	-
18	55 F	Low	2B	3	LCP 2.7 mm	LCP 2.0 mm	LCP 2.0 mm	16	85	130°	2.0	-
19	79 F	Low	2B	3	LCP 2.7 mm	LCP 2.0 mm	-	23	65	95°	2.0	-
20	53 F	Low	2B	2	LCP 2.7 mm	-	LCP 2.4 mm	63	65	90°	1.5	-
21	69 M	Low	2B	3	LCP 3.5 mm pelvic reconstruction plate	-	LCP 2.0 mm	23	100	-	1.5	-
22	53 F	Low	2B	3	LCP 2.7 mm	LCP 2.0 mm	LCP 2.0 mm	25	85	115°	1.5	10
23	70 F	Low	3B	4	LCP 2.0 mm	LCP 2.0 mm	-	-	-	95°	3.0	-
24	47 F	High	2B	4	LCP 2.7 mm	LCP 2.0 mm	LCP 2.0 mm	23	100	-	1.5	-
25	55 F	Low	3B	≥ 5	LCP 3.5 mm pelvic reconstruction plate	LCP 2.0 mm	LCP 2.0 mm	-	-	90°	1.5	-
26	28 M	High	3B	2	LCP 3.5 mm pelvic reconstruction plate	LCP 2.0 mm	-	-	-	135°	1.5	-

There were 17/26 isolated olecranon fractures. In the remaining nine cases, the olecranon fracture was accompanied by a radial head injury. Among these, two were Monteggia-like injuries; one patient had a Bado Type 3 fracture and a Mason Type 2 radial head fracture, and the other patient had a Bado Type 2 fracture and a Mason Type 3 radial head fracture.

The distribution of metaphyseal bone fragments varied: 6/26 patients had two fragments, 9/26 patients had three fragments, 4/26 patients had four fragments, and 7/26 patients had five or more fragments. The majority of fractures was classified as Mayo II-B (17/26) and III-B (8/26). Open fractures were observed in 5/26 patients (Grade I: *n* = 3, Grade II, *n* = 2) [14].

The posterior 2.7–3.5 mm LCP was combined with four different constructs: a unilateral medial LCP in 8/26 patients; a unilateral lateral LCP in 7/26; bilateral LCPs in 10/26; and bilateral with two lateral LCPs and one medial LCP in one patient.

### Clinical outcome measures (*n* = 26)

All fractures healed without the need for revision surgery at a median of 2.0 months (IQR 1.5–3.8) after the surgery. Range of motion was described for 24/26 patients at the final follow-up. The median flexion was 120° (IQR 110–130°) and the median extension-deficit 15° (IQR 10–21°), resulting in a flexion arc of 108° (IQR 94–116°). The median pronation was 88° (IQR 80–90°), and supination 85° (IQR 80–90°), resulting in a median forearm rotation arc of 168° (IQR 160–180°).

Hardware was electively removed in 7/26 patients because of local irritation after a median of 10 months (IQR 8–11). Complications occurred in 2/26 patients. One patient (patient 3) had an infection 2.5 years after the index surgery that resolved after hardware removal and antibiotic treatment. One patient (patient 9) had a re-fracture after a wrong movement one month after hardware removal. The fracture was treated with a lateral LCP, a 3.5 mm pelvic reconstruction plate and a radial prosthesis. After 21 months, the patient elected to remove the hardware of the revision surgery due to local irritation; and after two weeks, a re-re-fracture occurred that was treated with revision surgery again. The fracture healed three months later.

### Patient-reported outcome measures (*n* = 23)

The median Q-DASH score was 9 (IQR 0–22). Seven patients had the best possible score, meaning they did not experience any disability. The median MEPS was 90 (IQR 80–100) and 10/26 patients reported the best possible score. According to the MEPS, 12/26 patients had an excellent result, 6/26 had a good result, and 3/26 had a fair result.

## Discussion

The objective for surgical management of olecranon fractures is to obtain a congruent articular surface and a stable elbow to enable early postoperative rehabilitation. Obtaining anatomic reduction and stable fixation can be challenging if there is metaphyseal comminution and no obvious interdigitation between the large proximal fragment and the shaft. We describe a series of 26 patients with complex olecranon fractures with metaphyseal comminution that were treated with “orthogonal” plate fixation: a small 2.0–2.4 mm lateral and/or medial LCP combined with a contoured 3.5 mm–2.7 mm posterior LCP. With these small plate(s), the comminuted metaphyseal area is rebuilt first, prior to placing the posterior plate. The step-wise anatomic read and subsequent fixation without additional stripping of metaphyseal fragments improves intrinsic stability and facilitates the reconstruction of the articular surface. Final fixation and compression is then provided by the posterior plate. This is different from the anatomic low profile plates used by others that have only screws going transversely (perpendicular to the longitudinal axis) or slightly oblique rather than having a compression force in the axial plane [15–18]. Our technique prevents the potential risk of failure due to extension/flexion forces at the fracture site. The screws we use medially and laterally are small (2.0/2.4 mm) compared to other systems, preserving bone stock. Our data show an excellent healing rate and good clinical and patient-reported outcomes.

We use an orthogonal plate construct consisting of a small 2.0–2.4 mm medial and/or lateral LCP and a contoured posterior 2.7–3.5 mm LCP. This strategy has several advantages. First, rebuilding the comminuted metaphyseal area with a small lateral or medial plate reduces the number of fragments before placing the posterior LCP. This facilitates reduction and better containment of the fragments. Second, the construct “covers” fracture lines in the sagittal and the coronal plane. This reduces fracture gap size, and (micro)movement which benefits the healing process. Third, the location of these small plates can easily be adjusted based on the fracture lines. When using an anatomic plate the location of the screw holes may be suboptimal in relation to the fracture lines. Fourth, from an economic standpoint the total costs for our plates and screws used are roughly about 60% lower as compared to certain anatomic plates.

For absolute stability in a periarticular location, we think that the biomechanical benefits of orthogonal plating outweigh the disadvantages of additional soft tissue dissection. Hoelscher-Doht et al., demonstrated that orthogonal plating with a small additional plate (similar as our technique) resulted in higher stiffness and yield load (the maximum load before permanent deformation occurs) compared to single dorsal LCP plating [19]. The fracture model in their study represented an extra-articular proximal ulna fracture, which is a different kind of fracture with other biomechanical consequences for fixation, as it does not involve the joint, compared to the vast majority of patients in our series described herein.

The PROMs of our series (Q-DASH: 9 [IQR 0–22] and MEPS: 90 [IQR 80–100]) can be compared to others. Morwood et al. treated 14 olecranon fractures with an intra-articular sagittal plane split with orthogonal fixation [5]. They reported a mean DASH score of 7 which is comparable to our score. Ellwein et al. treated 37 patients with low-profile double plating and 42 patients with a single dorsal LCP [20]. Their fractures were relatively simple, being a transverse olecranon fracture (AO type 21-B1; Mayo Type 1 A). There were comparable DASH scores; and only small differences in MEPS: 95 points for low-profile double plating; and 99 for the single LCP. The final range of motion in our patients (median flexion: 120°, extension-deficit: 15°, pronation: 88°, supination: 85°) is within the range of other studies [5, 16, 20, 21, 22]. Previous studies have dictated that this range of motion allows for most activities of daily living to be performed, which is supported by the relatively favorable PROMs in our series [23, 24].

Dorsal plating for olecranon fractures has been criticized for high hardware removal rates due to local irritation and impingement. Overall, hardware removal rates differ substantially between studies (0% and 50%) [15, 16, 18, 21]. It is known that personal factors play an important role in determining hardware removal [25]. In our series, 7/26 patients elected hardware removal. This is lower than in our previously reported series using the single dorsal plating technique (56%) and suggests that the additional medial and/or lateral plate is relatively well tolerated [26].

In our series 2/26 patients had a complication that required a reoperation: one late infection; and one refracture after hardware removal. Bouchard et al. evaluated 321 patients with an olecranon fracture (53% comminuted) that were treated with precontoured plate fixation [27]. They reported hardware failure in 17 patients (5.3%) and infection in 9 (2.8%); confirming the relative safety of our approach.

The present study has limitations. First, there are methodological shortcomings of a retrospective case series, such as the lack of pre-defined follow-up variables. However, we collected important clinical follow-up variables at a median follow-up of 27 months and patient reported outcomes of 88% of patients at a median follow-up of 38 months, which surpasses the majority of similar studies. Second, this is a single institution and single surgeon series with a relatively small sample size, which may limit generalizability. However, the clear surgical description should enable other surgeons to perform this procedure as well. Third, we did not include a matched cohort to compare our technique to other techniques (e.g., bilateral plating, single posterior plating). However, we used orthogonal plating specifically in fracture patterns in which we felt other techniques would have been insufficient and therefore such a comparison would have been strongly biased by the fracture pattern. Fourth, there was heterogeneity in fracture patterns and associated injuries, which makes comparisons with other studies difficult. However, we believe this displays the “real life” heterogeneity in complex olecranon fractures and provides broader support for our technique. Fifth, six patients originally reported the full DASH score and 17 patients the abbreviated Q-DASH score. The Q-DASH uses 11 items from the original 30-item DASH questionnaire and has been validated [6]. We therefore deducted the Q-DASH score in these six patients from the DASH score for uniformity.

In conclusion, orthogonal plating with a posterior 3.5 mm–2.7 mm LCP and a small medial and/or lateral LCP is a safe, relatively cheap and effective technique that leads to excellent healing rates and good clinical and patient-reported outcomes in complex, multi-fragmentary olecranon fractures with metaphyseal comminution.
